# Supplementary material for: Factors associated with dietary diversity among pregnant women in the western hill region of Nepal: A community based cross-sectional study
Source: PLoS One. 2021 Apr 8;16(4):e0247085. doi: 10.1371/journal.pone.0247085 (PMC8031299; doi:10.1371/journal.pone.0247085)
Supplement: S1 Table — (DOCX) [file pone.0247085.s001.docx]

**S1 Table** Food groups to construct Minimum Dietary Diversity for women of reproductive age

| **S.N.** | **Food groups** | **Examples** |
| --- | --- | --- |
| 1 | Grains, white roots and tubers and plantains | Corn/maize, rice, wheat, sorghum, millet, porridge, bread or their foods made from grains  White potatoes, white yams, white cassava or other foods made from roots |
| 2 | Pulses (beans, peas, and lentils) | Mature beans or peas, lentils or bean |
| 3 | Nuts and seeds | Tree nut, groundnut/peanut or certain seeds |
| 4 | Dairy | Milk, cheese, yogurt or other milk products |
| 5 | Meat, poultry, and fish | Liver, kidney, heart or other organ meats or blood-based foods  Beef, pork, lamb, goat, rabbit, chicken, duck  Fresh or dried fish, shellfish or seafood |
| 6 | Eggs | Eggs from poultry or any other bird |
| 7 | Dark green leafy vegetables | Dark green leafy vegetables including wild forms locally available vitamin A rich leaves such as amaranth, kale, spinach, cassava leaves |
| 8 | Other vitamin A-rich fruits and vegetables | Pumpkin, carrots, squash or sweet potatoes that are yellow or orange inside  Ripe mango, ripe papaya, dried peach |
| 9 | Other vegetables | Other vegetables (e.g. tomato, onion) and other locally available vegetables |
| 10 | Other fruits | Other fruits, including wild fruits and 100 % fruit juice made from these |
