# Supplementary material for: Factors associated with dietary diversity among pregnant women in the western hill region of Nepal: A community based cross-sectional study
Source: PLoS One. 2021 Apr 8;16(4):e0247085. doi: 10.1371/journal.pone.0247085 (PMC8031299; doi:10.1371/journal.pone.0247085)
Supplement: S2 Table — (DOCX) [file pone.0247085.s002.docx]

**S2 Table:** Variables included in assessing knowledge on nutrition during pregnancy

| **S.N.** | **Knowledge type** | **Categories** |
| --- | --- | --- |
| 1. | Knowledge on increasing amount of food during pregnancy | More food  Less food  same as during non-pregnancy |
| 2. | Knowledge on food source for iron | Meat, liver and fish  Fruits and Vegetables  Don’t know |
| 3. | Knowledge on food source for calcium | Milk and milk products  Seeds/vegetables  Don’t know |
| 4. | Knowledge on using iodized salt during pregnancy | Open/ Dhikke salt  Iodized salt  Don’t know |
| 5. | Knowledge on duration of iron supplementation | 6 month  3 month  Don’t know |
| 6. | Knowledge on fetal complication of maternal undernutrition | No  Yes |
| 7. | Knowledge on maternal complication of maternal undernutrition | No  Yes |
| 8. | *Knowledge on effect of maternal undernutrition on maternal health | Anemia  Preeclampsia  Risk of infection  Risk of death  Don’t know |
| 9. | Knowledge on effect of maternal under nutrition on fetal weight | Low birth weight and still birth  No effect on fetal weight  Don’t know |

*Multiple choices
