# Supplementary material for: Factors associated with dietary diversity among pregnant women in the western hill region of Nepal: A community based cross-sectional study
Source: PLoS One. 2021 Apr 8;16(4):e0247085. doi: 10.1371/journal.pone.0247085 (PMC8031299; doi:10.1371/journal.pone.0247085)
Supplement: S3 Table — (DOCX) [file pone.0247085.s003.docx]

**S3 Table: Variables included in construction of women empowerment index**

| **S.N.** | **Indicators** | **Categories** | **Scoring** |
| --- | --- | --- | --- |
| 1. | Woman’s involvement in household decision-making | - access to health care, - household purchasing, and - freedom to visit relatives. | - participated in all three decisions- “2” score; - participated in one or two decisions-“1” score; and - did not participate in any decisions- “0” score |
| 2. | Woman’s membership in community groups | - a member of any community groups, such as a mothers’ group (aamasamuha), saving group (bachat samuha), women’s group (mahila samuha) and others, - not involved in any groups | - a member of any community groups-“1” score - not involved in any groups-“0” score |
| 3. | Woman’s cash earnings. | - earned cash only or both cash and in-kind, - did not earn cash at all | - earned cash only or both cash and in-kind-“1” score - did not earn cash at all-“0” score |
| 4. | Woman’s ownership of house/land | - owned a house, land, or both alone or jointly with husband - did not own a house, land or both | - owned a house, land, or both alone or jointly with husband-“1” score - did not own a house, land or both-“0” score |
| 5. | Woman’s education | - Attend secondary or higher education - Attend primary level education - Did not attend school | - attend secondary or higher education-“2” score - attend primary level education-“1” score - did not attend school-“0” score |
