# Supplementary material for: Factors associated with dietary diversity among pregnant women in the western hill region of Nepal: A community based cross-sectional study
Source: PLoS One. 2021 Apr 8;16(4):e0247085. doi: 10.1371/journal.pone.0247085 (PMC8031299; doi:10.1371/journal.pone.0247085)
Supplement: S4 Table — (DOCX) [file pone.0247085.s004.docx]

**S4 Table: VIF report of predictor variables**

| **S.N.** | **Variables** | **VIF** |
| --- | --- | --- |
| 1. | Ethnicity | 1.90 |
| 2. | Family type | 1.17 |
| 3. | Participant education level | 1.40 |
| 4. | Participant’s partner education | 1.35 |
| 5. | Participant employment | 1.06 |
| 6. | Wealth quintile index | 1.26 |
| 7. | Land ownership | 1.42 |
| 8. | Women empowerment | 1.55 |
| 9. | ANC visits | 1.03 |
| 10. | Food taboos | 1.05 |
| 11. | Nutritional knowledge | 1.12 |
