# Supplementary material for: Factors associated with dietary diversity among pregnant women in the western hill region of Nepal: A community based cross-sectional study
Source: PLoS One. 2021 Apr 8;16(4):e0247085. doi: 10.1371/journal.pone.0247085 (PMC8031299; doi:10.1371/journal.pone.0247085)
Supplement: S1 File — (DOCX) [file pone.0247085.s005.docx]

## Annex III: Questionnaire in English

Title: Factors associated with dietary diversity among pregnant women in the western hill region of Nepal: a community based cross-sectional study

| Instruction: Please tick the appropriate answer and fill in the blank where appropriate. | | | | | | | | | | | | | | | | | | | |  |  |
| --- | --- | --- | --- | --- | --- | --- | --- | --- | --- | --- | --- | --- | --- | --- | --- | --- | --- | --- | --- | --- | --- |
| ID NO: | | | | | | | | Date of interview: | | | | | | | | | | | |  |  |
| Ward no.: | | | | | | | | Time of interview: am/pm | | | | | | | | | | | |  |  |
| Q.N. | | Question | | | Response | | | | | | Code | | | | | | Skip/Remarks | | | | |
| A. **Socio-demographic information** | | | | | | | | | | | | | | | | | | | | | |
| A1 | | What is your name and cast/ethnicity? | | |  | | | | | |  | | | | | |  | | | | |
| A2 | | What is your age? | | |  | | | | | |  | | | | | |  | | | | |
| A6 | | What is your religion? | | | ☐ Hindu  ☐ Buddhist  ☐ Islam  ☐ Christian  ☐ Others…. | | | | | | 1  2  3  4  99 | | | | | |  | | | | |
| A7 | | Did you ever attend school? | | | ☐ Yes  ☐ No | | | | | | Yes=1  No=0 | | | | | |  | | | | |
| A8 | | What is the highest level of your education you have completed? | | | …………………………. | | | | | |  | | | | | |  | | | | |
| A9 | | What is the highest level of education your husband/partner has completed? | | | ……………………… | | | | | |  | | | | | |  | | | | |
| A10 | | What is your occupation, that is, what kind of work do you mainly do? | | | ☐ Agriculture  ☐ Service  ☐ Business  ☐ Labour work  ☐ Housewife  ☐ Other (specify)…... | | | | | | 1  2  3  4  5  99 | | | | | |  | | | | |
| A11 | | Earning status of the women | | | ☐ Cash only  ☐ In-kind only  ☐ Both cash and in-kind  ☐ Not paid | | | | | | 1  2  3  4 | | | | | |  | | | | |
| A12 | | What is the major occupation of your husband/partner? | | | ☐ Agriculture  ☐ Service  ☐ Business  ☐ Labour work  ☐ Foreign employment  ☐ Other (specify)…... | | | | | | 1  2  3  4  5  99 | | | | | |  | | | | |
| A13 | | How many members are there in your family? | | | ..................... | | | | | |  | | | | | |  | | | | |
| A14 | | What is your type of family? | | | ☐ Nuclear  ☐ Joint  ☐ Extended | | | | | | 1  2  3 | | | | | |  | | | | |
| A15 | | Who is the household head of your family? | | | ☐ Male  ☐ Female | | | | | | 1  2 | | | | | |  | | | | |
| **B. Pregnant Women health related information** | | | | | | | | | | | | | | | | | | | | | |
| B1 | | How many of times have you been pregnant (including current pregnancy) | | | ………………………… | | | | | |  | | | | | |  | | | | |
| B2 | | Number of miscarriages: | | | ................................... | | | | | |  | | | | | |  | | | | |
| B3 | | Number of Children: | | | ................................... | | | | | |  | | | | | |  | | | | |
| B4 | | Last menstrual period: | | | ............................... | | | | | |  | | | | | |  | | | | |
| B5 | | Did you receive ANC checkup for your pregnancy? | | | ☐Yes  ☐ No | | | | | | Yes=1  No=0 | | | | | |  | | | | |
| B6 | | If yes, how many months pregnant were you when you first received antenatal care for this pregnancy | | | ………………  Don’t know | | | | | |  | | | | | |  | | | | |
| B7 | | If yes, how many times did you receive ANC for your pregnancy? | | | ☐ 1  ☐ 2  ☐ 3  ☐ 4  ☐ >4 | | | | | |  | | | | | |  | | | | |
| B8 | | Are you currently taking iron tablets? | | | ☐ Yes  ☐ No | | | | | | 1  0 | | | | | |  | | | | |
| B9 | | Are you currently taking a multivitamin tonic (energy bottle)? | | | ☐ Yes  ☐ No | | | | | | 1  0 | | | | | |  | | | | |
| **C.** **Socio-economic information** | | | | | | | | | | | | | | | | | | | | |  |
| C1 | | Does your household have?  A television  A mobile telephone?  A landline telephone?  A refrigerator?  A bed?  A sofa?  A cupboard?  A computer?  A fan?  A clock  A dhiki/janto | | | ☐ Yes ☐ No  ☐ Yes ☐ No  ☐ Yes ☐ No  ☐ Yes ☐ No  ☐ Yes ☐ No  ☐ Yes ☐ No  ☐ Yes ☐ No  ☐ Yes ☐ No  ☐ Yes ☐ No  ☐ Yes ☐ No  ☐ Yes ☐ No | | | | | Yes=1  No=0 | | | | |  | | | | | |  |
| C2 | | What type of fuel does your household mainly use for cooking? | | | ☐ Electricity  ☐ LPG  ☐ Biogas  ☐ Kerosene  ☐ Wood  ☐ Animal dung  ☐ Others….(specify) | | | | | 1  2  3  4  5  6  99 | | | | |  | | | | | |  |
| C3 | | Do you have a separate room, which is used, as a kitchen? | | | ☐ Yes  ☐ No | | | | | 1  0 | | | | |  | | | | | |  |
| C4 | | Main material of the floor | | | ☐ Earth/dung  ☐ Cemented  ☐ Ceramic tiles  ☐ Finished floor/parquet  ☐ Other (specify)………… | | | | | 1  2  3  4  99 | | | | | Observe and write | | | | | |  |
| C5 | | Main material of the roof | | | ☐ Thatched roof  ☐ Galvanized sheet  ☐ Ceramic tiles  ☐Cement  ☐ Other (specify)………. | | | | | 1  2  3  4  99 | | | | | Observe and write | | | | | |  |
| C6 | | Main materials of the exterior walls | | | ☐Tin  ☐Mud and stone/brick  ☐Wood/bamboo  ☐Concrete  ☐Other (specify)………… | | | | | 1  2  3  4  99 | | | | | Observe and write | | | | | |  |
| C7 | | How many rooms in this household are used for sleeping? | | | ……..rooms | | | | |  | | | | |  | | | | | |  |
| C8 | | Does any member of this household own  A bicycle/ rickshaw?  A motorcycle or motor scooter?  A three wheel tempo?  A tractor?  A car or truck? | | | ☐ Yes ☐ No  ☐ Yes ☐ No  ☐ Yes ☐ No  ☐ Yes ☐ No  ☐ Yes ☐ No | | | | | Yes=1  No=0 | | | | |  | | | | | |  |
| C9 | | Does any member of this household own any agricultural land? | | | ☐ Yes  ☐ No | | | | | 1  0 | | | | | If 0= skip to Q.N. C11 | | | | | |  |
| C10 | | If yes, how many aana/ropani of agricultural land do members of this household own? | | | ☐ Aana…………  ☐ Ropani………. | | | | | 1  2 | | | | |  | | | | | |  |
| C11 | | Does this household own any livestock, herds, other farm animals or poultry? | | | ☐ Yes  ☐ No | | | | | 1  0 | | | | |  | | | | | |  |
| C12 | | How many of the following animals does this household own? | | | ☐ Buffalo ………………  ☐ Cow and bull ………..  ☐ Goat …………………  ☐ Chicken/duck ………..  ☐ Pig ………………….. | | | | |  | | | | | If 0= skip to Q.N. C13 | | | | | |  |
| C13 | | Does your household own any kitchen garden? | | | ☐ Yes  ☐ No | | | | |  | | | | | If 0= skip to Q.N. D1 | | | | | |  |
| C14 | | What have you grown in your kitchen garden? | | | ....................  …………………………. | | | | |  | | | | |  | | | | | |  |
| C15 | | Does your kitchen garden product is sufficient for your family consumption? | | | ☐ Yes  ☐ No | | | | | 1  0 | | | | |  | | | | | |  |
| C16 | | In the past four weeks, how often did your household bought vegetables for consumption? | | | ☐ Never  ☐ Rarely  ☐ Sometimes  ☐ Often | | | | | 1  2  3  4 | | | | |  | | | | | |  |
| **D. Dietary diversity questionnaire** | | | | | | | | | | | | | | | | | | | | | |
| Now I’d like to ask you to describe everything that you ate or drank yesterday and the day before yesterday during the day or night, whether you ate it at home or anywhere else. Please include all foods and drinks, any snacks or small meals, as well as any main meals. Remember to include all foods you may have eaten while preparing meals or preparing food for others. Please also include food you ate even if it was eaten elsewhere, away from your home. Also specify the portion of the each food you consumed. Let’s start with the first food or drink consumed yesterday | | | | | | | | | | | | | | | | | | | | | |
| D1 | | Did you have anything to eat or drink when you woke? | | | ☐ Yes  ☐ No | | | | | Yes=1  No=0 | | | | | | If 0= skip to Q.N. D3 | | | | | |
| D2 | | If yes, what and what was the estimated amount? Anything else?* | | | ………………………  ……………………….  ………………………... | | | | |  | | | | | |  | | | | | |
| D3 | | Did you have anything to eat or drink later in the morning? | | | ☐ Yes  ☐ No | | | | | 1  0 | | | | | | If 0= skip to Q.N. D5 | | | | | |
| D4 | | If yes, what and what was the estimated amount? Anything else?* | | | ………………………..  …………………………  ……………………….. | | | | |  | | | | | |  | | | | | |
| D5 | | Did you have anything to eat or drink during the afternoon? | | | ☐ Yes  ☐ No | | | | | 1  0 | | | | | | If 0= skip to Q.N. D7 | | | | | |
| D6 | | If yes, what and what was the estimated amount? Anything else?* | | | …………………………  …………………………..  ………………………….. | | | | |  | | | | | |  | | | | | |
| D7 | | Did you have anything to eat in the evening? | | | ☐ Yes  ☐ No | | | | | 1  0 | | | | | | If 0= skip to Q.N. D9 | | | | | |
| D8 | | If yes, what and what was the estimated amount? Anything else?* | | | ………………………….  ……………………………  …………………………... | | | | |  | | | | | |  | | | | | |
| D9 | | Did you have anything else to eat or drink in the evening before going to bed or during the night? | | | ☐ Yes  ☐ No | | | | | 1  0 | | | | | | If 0= skip to Q.N. 1.1 | | | | | |
| D10 | | If yes, what and what was the estimated amount? Anything else?* | | | ……………………………  …………………………….  …………………………… | | | | |  | | | | | |  | | | | | |
|  | Was last day a special day, like a celebration, feast day, fasting, sickness etc in which you ate special food or more or less than usual or did not eat because of fasting? | | | | | | | | How many times have you eaten ___________ in the past one day? | | | | | 0=No 1=Yes 9=Don’t know | | | | | | | |
| **E. Information about cultural practices** | | | | | | | | | | | | | | | | | | | | | |
|  | | | | Question | | Response | | | | | | | Code | | | | | Remarks/  skip | | | |
| E1 | | | | Are there any foods that are avoided since you became pregnant because of cultural beliefs and norms? | | ☐ Yes  ☐ No | | | | | | | Yes=1  No=0 | | | | | If 0= skip to Q.N. F1 | | | |
| E2 | | | | Which foods? (ASK NAMING FOODTYPE) | | …………………………  ………………………… | | | | | | |  | | | | |  | | | |
| E3 | | | | Why these foods are avoided for pregnancy? (probe the reason) | | ………………………..  ……………………….. | | | | | | |  | | | | |  | | | |
| **F. Knowledge on appropriate nutrition during pregnancy** | | | | | | | | | | | | | | | | | | | | | |
| F1 | | | How should a pregnant woman eat in comparison to a non-pregnant woman? | | | ☐Eat more food each day  ☐Eat more times each day  ☐Eat only what she craves  ☐The same as in non-pregnancy | | | | | | 1  2  3  4 | | | | | | | Multiple response | | |
| F2 | | | Have you heard about iron rich food? | | | ☐Yes  ☐No | | | | | | 1  0 | | | | | | | If 0= skip to Q.N. F4 | | |
| F3 | | | If yes, can you tell me the source of iron rich food? | | | …………………………………  ………………………………… | | | | | |  | | | | | | |  | | |
| F4 | | | How long should a woman consume iron supplement during pregnancy? | | | …………………………………. | | | | | |  | | | | | | |  | | |
| F5 | | | Have you heard about calcium rich food? | | | ☐Yes  ☐No | | | | | | 1  0 | | | | | | |  | | |
| F6 | | | If yes, can you tell me the source of calcium rich food? | | | ………………………………..  ………………………………… | | | | | |  | | | | | | |  | | |
| F7 | | | In your opinion, what type of salt a pregnant woman should consume? | | | ☐Open/ Dhikke salt  ☐Black salt  ☐Iodized salt  ☐Others  ☐Don’t know | | | | | | 1  2  3  4  5 | | | | | | |  | | |
| F8 | | | If a pregnant woman is undernourished, do you think there will be health risk to the women? | | | ☐Yes  ☐No | | | | | | 1  0 | | | | | | | If 0= skip to Q.N. F8 | | |
| F9 | | | If yes, what are the health risks for the women? | | | ☐Anemia  ☐Pre-eclampsia  ☐Risk of infection  ☐Risk of dying  ☐Others | | | | | | 1  2  3  4  5 | | | | | | | Multiple response | | |
| F10 | | | When a pregnant woman is undernourished do you think there will be health risk to the newborn? | | | ☐Yes  ☐No | | | | | | 1  0 | | | | | | | If 0= skip to Q.N. G1 | | |
| F11 | | | If yes, What do you think are the effect of maternal under nutrition on fetal weight? | | | ☐Low birth weight (small baby)  ☐Still birth  ☐No effect on fetal weight  ☐Don’t know | | | | | | 1  2  3  4 | | | | | | |  | | |
| **G.** **Information regarding household food security** | | | | | | | | | | | | | | | | | | | | | |
| G1 | | | | In the past four weeks, did you worry that your household would not have enough food? | | | ☐ Yes  ☐ No | | | | | | Yes=1  No=0 | | | | | If 0=skip to Q G2 | | | |
| G1.1 | | | | How often did this happen? | | | ☐ Rarely (1 -2 times)  ☐ Sometimes (3 -10 times)  ☐ Often (>10 times) | | | | | | 1  2  3 | | | | |  | | | |
| G2 | | | | In the past four weeks, were you or any household member not able to eat the kinds of foods you preferred because of a lack of resources? | | | ☐ Yes  ☐ No | | | | | |  | | | | | If 0=skip to Q.N. G3 | | | |
| G2.1 | | | | How often did this happen? | | | ☐ Rarely (1 -2 times)  ☐ Sometimes (3 -10 times)  ☐ Often (>10 times) | | | | | | 1  2  3 | | | | |  | | | |
| G3 | | | | In the past four weeks, did you or any household member have to eat a limited variety of foods due to a lack of resources? | | | ☐ Yes  ☐ No | | | | | |  | | | | | If 0=skip to Q.N. G4 | | | |
| G3.1 | | | | How often did this happen? | | | ☐ Rarely (1 -2 times)  ☐ Sometimes (3 -10 times)  ☐ Often (>10 times) | | | | | | 1  2  3 | | | | |  | | | |
| G4 | | | | In the past four weeks, did you or any household member have to eat some foods that you really did not want to eat because of a lack of resources to obtain other types of food? | | | ☐ Yes  ☐ No | | | | | |  | | | | | If 0=skip to Q.N. G5 | | | |
| G4.1 | | | | How often did this happen? | | | ☐ Rarely (1 -2 times)  ☐ Sometimes (3 -10 times)  ☐ Often (>10 times) | | | | | | 1  2  3 | | | | |  | | | |
| G5 | | | | In the past four weeks, did you or any household member have to eat a smaller meal than you felt you needed because there was not enough food? | | | ☐ Yes  ☐ No | | | | | |  | | | | | If 0=skip to Q.N. G6 | | | |
| G5.1 | | | | How often did this happen? | | | ☐ Rarely (1 -2 times)  ☐ Sometimes (3 -10 times)  ☐ Often (>10 times) | | | | | | 1  2  3 | | | | |  | | | |
| G6 | | | | In the past four weeks, did you or any other household member have to eat fewer meals in a day because there was not enough food? | | | ☐ Yes  ☐ No | | | | | |  | | | | | If 0=skip to Q.N. G7 | | | |
| G6.1 | | | | How often did this happen? | | | ☐ Rarely (1 -2 times)  ☐ Sometimes (3 -10 times)  ☐ Often (>10 times) | | | | | | 1  2  3 | | | | |  | | | |
| G7 | | | | In the past four weeks, was there ever no food to eat of any kind in your household because of lack of resources to get food? | | | ☐ Yes  ☐ No | | | | | |  | | | | | If 0=skip to Q.N. G8 | | | |
| G7.1 | | | | How often did this happen? | | | ☐ Rarely (1 -2 times)  ☐ Sometimes (3 -10 times)  ☐ Often (>10 times) | | | | | | 1  2  3 | | | | |  | | | |
| G8 | | | | In the past four weeks, did you or any household member go to sleep at night hungry because there was not enough food? | | | ☐ Yes  ☐ No | | | | | |  | | | | | If 0=skip to Q.N. G9 | | | |
| G8.1 | | | | How often did this happen? | | | ☐ Rarely (1 -2 times)  ☐ Sometimes (3 -10 times)  ☐ Often (>10 times) | | | | | | 1  2  3 | | | | |  | | | |
| G9 | | | | In the past four weeks, did you or any household member go a whole day and night without eating anything because there was not enough food? | | | ☐ Yes  ☐ No | | | | | |  | | | | | If 0= skip to Q.N. H1 | | | |
| G9.1 | | | | How often did this happen? | | | ☐ Rarely (1 -2 times)  ☐ Sometimes (3 -10 times)  ☐ Often (>10 times) | | | | | | 1  2  3 | | | | |  | | | |
| **H. Information on women’s empowerment** | | | | | | | | | | | | | | | | | | | | | |
| H1 | | Do you belong to any group? Please specify.  Mother’s group  Saving/ co-operative group Women’s group | | | | | ☐Yes ☐ No  ☐ Yes ☐ No  ☐ Yes ☐ No | | | | | | Yes=1  No=0 | | | | |  | | | |
| H2 | | Do you own this or any other house either alone or jointly with someone else? | | | | | ☐ Alone only  ☐ Jointly only  ☐ Both alone and jointly  ☐ Does not own | | | | | | 1  2  3  4 | | | | |  | | | |
| H3 | | Do you own any land either alone or jointly with someone else? | | | | | ☐ Alone only  ☐ Jointly only  ☐ Both alone and jointly  ☐ Does not own | | | | | | 1  2  3  4 | | | | |  | | | |
| H4 | | Who usually makes decisions about health care for yourself | | | | | ☐ Participant only  ☐ Husband only  ☐ Participant and husband jointly  ☐ Other (specify)…........... | | | | | | 1  2  3  99 | | | | |  | | | |
| H5 | | Who usually makes decisions about making major household purchases? | | | | | ☐ Participant only  ☐ Husband only  ☐ Participant and husband jointly  ☐ Other (specify)…........... | | | | | | 1  2  3  99 | | | | |  | | | |
| H6 | | Who usually makes decisions about visits to your family or relatives? | | | | | ☐ Participant only  ☐ Husband only  ☐ Participant and husband jointly  ☐ Other (specify)…........... | | | | | | 1  2  3  99 | | | | |  | | | |

## Annex VI : Questionnaire in Nepali

**u{ejlt dlxnfnfO{ ;f]lBg] k|ZgfjnL**

| lgb]{zgM s[kof ;lx pQ/df l6s √_ nufpg'xf];\ jf vfln 7fpFdf eg{'xf];\. | |
| --- | --- |
| ;+s]t g+=M | cGt/jftf{ lnPsf] ldlt -;fn÷dlxgf÷ut]_M |
| j8f g+=M | cGt/jftf{ z'? u/]sf] ;doM ============ah] -ljxfg÷a']nsL_ |

| Effu s M hg;f+lvs ljj/0f | | | | | | | | | | | | | | | |
| --- | --- | --- | --- | --- | --- | --- | --- | --- | --- | --- | --- | --- | --- | --- | --- |
| k\|Zg g+= | | | | k\|Zg | ljsNkx? | | | sf]8 | | | | | s}lkmot | | |
| s ! | | | | tkfO[sf] gfd / y/÷hft s] xf]< | ============= | | |  | | | | |  | | |
| s@ | | | | tkfO{ slt jif{sf] x'geof] < | =============jif{ | | |  | | | | | K'f/fePsf] jif{ | | |
| s# | | | | tkfO{ s'g wd{ dfGg' x'G5 < | ☐ lxGb"  ☐ af}4  ☐ d'lZnd  ☐ ls\|l:rog  ☐ cGo -pNn]v ug]{_================ | | | !  @  #  $  (( | | | | |  | | |
| s$ | | | | s] tkfO{ slxn] ljBfno hfg'ePsf] 5< | ☐ 5  ☐ 5}g | | | !  ) | | | | | olb 5}g eg] k\|Zg g=s^ ;f]Wg] | | |
| s% | | | | tkfO{n] pRrtd z}lIfs ofUotf slt ;Dd xfFl;n ug'{ ePsf] 5< | ============================================ | | |  | | | | |  | | |
| s^ | | | | tkfO{sf] >Ldfgn] pRrtd z}lIfs ofUotf slt ;Dd xfFl;n ug'{ ePsf] 5< | =============================================== | | |  | | | | |  | | |
| s& | | | | tkfO{sf] d'Vo k]zf s] xf]< | ☐ s[lif  ☐ hflu/  ☐ Jofkf/  ☐ b}lgs Hofnfbf/L  ☐ u[x0fL  ☐ cGo -pNn]v ug]{_================= | | | !  @  #  $  %  (( | | | | |  | | |
| s* | | | | s] tkfO{n] sfd u/] jfkt lhlG; jf yf]s sdfpg' x'G5< | ☐ lhlG; dfq sdfp5'  ☐ yf]s dfq sdfp5'  ☐ lhlG; / yf]s b'j} sdfp5'  ☐ s]lx sdfplbg | | | !  @  #  $ | | | | |  | | |
| s( | | | | tkfO{sf] >Ldfgsf] k]zf s] xf]< | ☐ s[lif  ☐ hflu/  ☐ Jofkf/  ☐ b}lgs Hofnfbf/L  ☐ j}b]lzs /f]huf/?  ☐ cGo -pNn]v ug]{_================= | | | !  @  #  $  %  (( | | | | |  | | |
| s!) | | | | kl/jf/ ;+Vof | ============= | | |  | | | | |  | | |
| s!! | | | | kl/jf/sf] k\|sf/ | ☐ Psn  ☐ ;+o"Qm  ☐ a[xt | | | !  @  # | | | | |  | | |
| s!@ | | | | tkfOsf] 3/df 3/d'lnsf] x'g'x'G5< | ☐ dlxnf  ☐ k'?if | | | !  @ | | | | |  | | |
| Effu v uef{jlt dlxnfsf] :jf:Yo ;DaGwL hfgsf/L | | | | | | | | | | | | | | | |
| v! | | | xfnsf] ue{ ;d]t u/]/ tkfO{ slt k6s ue{jtL aGg' eof] < | | =============================== | | |  | | | | |  | | |
| v@ | | | v]/ uPsf] ue{sf] ;+Vof, olb 5 eg], | | ========================================== | | |  | | | | |  | | |
| v# | | | jRrfsf] ;+Vof, olb 5g\eg | | ========================================= | | |  | | | | |  | | |
| v$ | | | olb o; eGbf klxnf ue{jtL x'g' ePsf] lyof] eg], b'O{ j6f ue{ ljrsf] cGt/ slt lyof]< | | ===================================== | | |  | | | | |  | | |
| v% | | | clGtd k6s dlxgfjf/L slxn] ePsf] lyof] < | | ============================================= | | |  | | | | |  | | |
| v^ | | | s] tkfO{n] cfkmgf\] uef{jf:yfsf]nflu ue{jlt hfr u/fpg'ePsf] 5< | | ☐ 5  ☐ 5}g | | | !  ) | | | | | olb 5}g eg] k\|Zg g= v ( ;f]Wg] | | |
| v& | | | olb 5 eg] tkfO{n] klxnf]k6s hfr ubf{ tkfOsf] sltcf} dlxgf rln/xsf] lyof]< | | ====================================== | | |  | | | | |  | | |
| v* | | | Xfn;Dd tkfO{n] sltk6s ue{hfr ug{'ePsf] 5< | | ☐ !  ☐ @  ☐ #  ☐ $  ☐ > $ | | |  | | | | |  | | |
| v( | | | s] tkfO{n] xfn cfO/g rSsL vfO{/xg' ePsf] 5 < | | ☐ 5  ☐ 5}g | | | !  ) | | | | |  | | |
| v!) | | | s] tkfO{n] xfn s'g} le6fldg vfO{/xg' ePsf] 5 < | | ☐ 5  ☐ 5}g | | | !  ) | | | | |  | | |
| Effu u M;fdflhs cfly{s ljj/0f | | | | | | | | | | | | | | | |
| u! | | | s] tkfO{sf] 3/df lgDg s'/fx? 5g\<  lah'nL  /]l8of]  l6eL  df]afOn  kmf]g  6]lnkmf]g  lk\|mh  6]a'n  s'rL{  vf6  ;f]kmf  b/fh  sDKo'6/  38L  k+vf  l9sL]÷hfFtf | | ☐ 5 ☐ 5}g  ☐ 5 ☐ 5}g  ☐ 5 ☐ 5}g  ☐ 5 ☐ 5}g  ☐ 5 ☐ 5}g  ☐ 5 ☐ 5}g  ☐ 5 ☐ 5}g  ☐ 5 ☐ 5}g  ☐ 5 ☐ 5}g  ☐ 5 ☐ 5}g  ☐ 5 ☐ 5}g  ☐ 5 ☐ 5}g  ☐ 5 ☐ 5}g | | | 5 Ö!  5}gÖ) | | | | |  | | |
| u@ | | | tkfO{sf] 3/df vfgf ksfpg w]/}h;f] s] k\|of]u ug'{x'G5< | | ☐ lah'nL  ☐ Pn=lk=Uof;  ☐ uf]a/ Uof;  ☐ dl§t]n  ☐ bfp/f  ☐ uf]a/sf] u'O7f]  ☐ cGo -pNn]v ug]{_============= | | | !  @  #  $  %  ^  (( | | | | |  | | |
| u# | | | s] tkfO{sf] 3/df efG5f sf]7f 56\6} 5< | | ☐ 5  ☐ 5}g | | | !  ) | | | | |  | | |
| u$ | | | tkfO{sf] 3/sf] e'OF s]n] ag]sf] 5< | | ☐ Dff6f]÷uf]a/  ☐ l;d]G6  ☐ dfa{n÷6fon  ☐ kfs]{l6Ë ul/Psf]  ☐ cGo -pNn]v ug]{_============= | | | !  @  #  $  (( | | | | | cjnf]sg ug{ | | |
| u% | | | tkfO{sf] 3/sf] 5fgf s]n] ag]sf] 5< | | ☐ k/fn÷:ofpnf  ☐ h:tf kftf  ☐ 6fon  ☐ l;d]G6  ☐ cGo -pNn]v ug]{_============= | | | !  @  #  $  (( | | | | | cjnf]sg ug{ | | |
| u^ | | | tkfO{sf] 3/sf] leTtf s]n] ag]sf] 5< | | ☐ l6g  ☐ Dff6f] / 9'+uf÷O{6\6f  ☐ sf7÷aFf;  ☐ l;d]G6  ☐ cGo -pNn]v ug]{_================== | | | !  @  #  $  (( | | | | | cjnf]sg ug{ | | |
| u& | | | tkfO{sf] 3/df ;'Tgsf] nflu sltj6f sf]7f Kf\|of]u ug'{x'G5< | |  | | |  | | | | |  | | |
| u* | | | s] tkfO{sf] kl/jf/sf] s'g} klg ;b:ox? ;Fu lgDg s'/fx? 5g\<  ;fO{sn  df]6/ ;fO{sn÷:s'6/  6]Dkf]  v]tdf k\|of]ux'\g] 6\ofS6/  sf/÷6«s | | ☐ 5 ☐ 5}g  ☐ 5 ☐ 5}g  ☐ 5 ☐ 5}g  ☐ 5 ☐ 5}g  ☐ 5 ☐ 5}g | | | 5 Ö!  5}gÖ) | | | | |  | | |
| u( | | | s] tkfO{sf] 3/df cfˆgf] v]tLof]Uo hUuf÷hldg 5< | | ☐ 5  ☐ 5}g | | | !  ) | | | | | olb 5}g eg] k\|Zg g=u!) ;f]Wg] | | |
| u(=! | | | olb 5 eg],slt 5< | | ☐ cfgf================  ☐ /f]kgL=============== | | |  | | | | |  | | |
| u!) | | | s] tkfO{sf] 3/df kz'kfng ug'{ePsf] 5< | | ☐ 5  ☐ 5}g | | | !  ) | | | | | olb 5}g eg] k\|Zg g=u!! ;f]Wg] | | |
| u!)=! | | | tkfO{sf] 3/df pNn]v ul/Psf] 3/kfn'jf rf}kfof sltj6f 5g\< | | ☐ e};L ==============  ☐ ufO{÷uf]? ==============  ☐ afv\|f ==============  ☐ s'v'/f÷xfF;==============  ☐ ;'+u'/ | | | !  @  #  $  (( | | | | |  | | |
| Uf!! | | | s] tkfO{sf] 3/df s/];faf/L 5 < | | ☐ 5  ☐ 5}g | | |  | | | | | olb 5}g eg] k\|Zg g=3! ;f]Wg] | | |
| Uf!!=! | | | tkfO{n] s/];faf/L s] s] pTkfbg ug'{ePsf] 5 < | | =================================  ================================== | | |  | | | | |  | | |
| Uf!!=@ | | | s] s/];faf/Lsf] pTkfbg tkfO{sf] kl/jf/sf] b}lgs pkof]usf nfuL k\|ofKt x'G5 < | | ☐x'G5  ☐ x'b}}g | | |  | | | | |  | | |
| Uf!!=# | | | ut rf/ xKtfdf, tkfO{sf] kl/jf/n] k\|foM h;f] slt t/sf/L lsGg' ePsf] lyof] xf]nf< | | ☐ slxNo}  ☐ lj/n}  ☐ slxn] sflx  ☐ k\|foh;f] | | |  | | | | |  | | |
| Vf08 3 VffBo ljljBtfsf] ljlj/0f | | | | | | | | | | | | | | | |
| s[kof ca d tkfO{nfO{ lxhf] laxfg b]lv /ftL ;Dd vfg' ePsf] vfg]s'/f jf lkpg] s'/fx? ;a}sf] gfd atfO{lbg'xf]nf, rfx] Tof] 3/df vfPsf] xf]; jf 3/ aflx/ vfhfsf] ?kdf xf]; . laxfg p7\g] lalQs} vfg'ePsf] jf lkpg' ePsf] lrhaf6 ;'? ug'{xf]; | | | | | | | | | | | | | | | |
| 3! | | tkfO{ lxhf] ljxfg p7] nut} s]xL vfgf jf t/n vfg' ePsf] lyof]< olb lyof] eg] s] s] lyof] < | | | ☐ lyof]  ☐ lyPg | | | | !  ) | | | | | olb lyPg eg] k\|Zg g= 3# ;f]Wg] | |
| 3@ | | olb lyof] eg] s] s] lyof] < -lj:t[tdf n]Vg'xf]; dfqf;lxt_ | | | ===========================================  ===============================================  ============================================== | | | |  | | | | |  | |
| 3# | | tkfO{ lxhf] ljxfg vfhfsf] ?kdf s]xL vfgf jf t/n vfg' ePsf] lyof]< olb lyof] eg]s] s] lyof] < | | | ☐ lyof]  ☐ lyPg | | | | !  ) | | | | | olb lyPg eg] k\|Zg g= 3% ;f]Wg] | |
| 3$ | | olb lyof] eg] s] s] lyof] < -lj:t[tdf n]Vg'xf]; dfqf;lxt _ | | | ==================================================  ==================================================  ================================================ | | | |  | | | | |  | |
| 3% | | tkfO{ lxhf] dWofGx jf lbp;f] vfgfsf] ?kdf s]xL vfgf jf t/n vfg' ePsf] lyof]< olb lyof] eg] s] s] lyof] < | | | ☐ lyof]  ☐ lyPg | | | | !  ) | | | | | olb lyPg eg] k\|Zg g= 3& ;f]Wg] | |
| 3^ | | olb lyof] eg] s] s] lyof] < -lj:t[tdf n]Vg'xf]; dfqf;lxt _ | | | =================================================  =====================================================  ==================================================== | | | |  | | | | |  | |
| 3& | | tkfO{ lxhf] ;fFem kv vfhfsf] ?kdf s]xL vfgf jf t/n vfg' ePsf] lyof]< olb lyof] eg] s] s] lyof] < | | | ☐ lyof]  ☐ lyPg | | | | !  ) | | | | | olb lyPg eg] k\|Zg g= 3( ;f]Wg] | |
| 3* | | olb lyof] eg] s] s] lyof] < -lj:t[tdf n]Vg'xf]; dfqf;lxt _ | | | =================================================  =================================================  ================================================== | | | |  | | | | |  | |
| 3( | | tkfO{ lxhf] /flt jf ;'Tg' c3f8L vfgf jf vfhfsf] ?kdf s]xL vfgf jf t/n vfg' ePsf] lyof]< olb lyof] eg] s] s] lyof] < | | | ☐ lyof]  ☐ lyPg | | | | !  ) | | | | | olb lyPg eg] k\|Zg g= !=! ;f]Wg] | |
| 3!) | | olb lyof] eg] s] s] lyof] < -lj:t[tdf n]Vg'xf]; dfqf;lxt _ | | | =================================================  ==============================================  =============================================== | | | |  | | | | |  | |
| 3!@ | ut @$ 306f s'g} k\|sf/n] ljz]if /x\of] ls h:t} rf8kj{, ef]h, jt{ jf lj/fdL h;df tkfO{n] ;flassf] eGbf sd jf w]/} vfgf vfg' ePsf] lyof] < | | | | | | ut @$ 306fdf slt k6s ==========pQm vfgf vfg' ePsf] lyof]< | | | | | ) lyPg  ! lyof]  ( yfxf ePg | | | |
| ª= vfgkfg ;Fu ;DalGwt ;fF:s[lts rfnrnsf] ljj/0f | | | | | | | | | | | | | | | |
| ª=! | | | To:tf s'g} vfg] s'/fx? 5g\h'g tkfO{n] tkfO{sf] dfGotf jf ljZjf;sf sf/0f vfFbf ue{nfO{ kmfO{bf ub{5 < | | | ☐ 5 ☐ 5}g | | | | 5 Ö!  5}gÖ) | | | | | olb 5}g eg] k\|Zg g= ª $ ;f]Wg] |
| ª=@ | | | olb 5g\eg] tL s'g s'g x'g\< | | | ============================================  ================================================ | | | |  | | | | |  |
| ª=# | | | pQm vfg] s'/fx? lsg jf s;/L ue{jtLsf nfuL /fd\|f] x'G5g\<  -vf]tNg'xf];_ | | |  | | | |  | | | | |  |
| ª=$ | | | To:tf s'g} vfg] s'/fx? 5g\h'g tkfO{n] tkfO{sf] dfGotf jf ljZjf;sf sf/0f vfFbf ue{nfO{ a]kmfO{bf ub{5 < | | | ☐ 5 ☐ 5}g | | | |  | | | | | olb 5}g eg] k\|Zg g=r! ;f]Wg] |
| ª=% | | | olb 5g\eg] tL s'g s'g x'g\< | | | ==============================================  ================================================= | | | |  | | | | |  |
| ª=^ | | | pQm vfg] s'/fx? lsg jf s;/L ue{jtLsf nfuL xfgLsf/s x'G5g\< -vf]tNg'xf];_ | | |  | | | |  | | | | |  |
| Rf Ufe{jtL cj:yfdf pko'tm vfgfsf] 1fg | | | | | | | | | | | | | | | |
| Rf! | | | Ufe{jtL gePsf] \dlxnfsf] t'ngfdf Ufe{jtL dlxnfn] s;/L vfg' k5{< | | ☐x/]s k6s B]/} vfgf vfg]  ☐ x/]s k6s B]/} vfgf vfg]  ☐rfx]sf] s'/fdfq vfg]  ☐Ps;dfg vfg] | | | | | | !  @  #  $ | | | | ax' pQ/ |
| Rf@ | | | s] tkfO{n] cfO/go'Qm vfgfsf]af/] ;'Gg' ePsf] 5< | | ☐ 5 ☐ 5}g | | | | | | 5 Ö!  5}gÖ) | | | | olb 5}g eg] k\|Zg g=r$ ;f]Wg] |
| Rf# | | | olb 5 eg] cfO/go'Qm vfgf s] s] x'g< | | ======================================= | | | | | |  | | | |  |
| Rf$ | | | Ufe{jtL dlxnfn] cfO/g rSsL slt nfdf] ;do vfg' k5{< | | ============================================== | | | | | |  | | | |  |
| Rf% | | | s] tkfO{n] Sofnl;odo'tm vfgfsf]af/] ;'Gg' ePsf] 5< | | ☐ 5 ☐ 5}g | | | | | | 5 Ö!  5}gÖ) | | | | olb 5}g eg] k\|Zg g= r& ;f]Wg] |
| Rf^ | | | olb 5 eg] Sofnl;odo'tm vfgf s] s] x'g\< | | ================================================ | | | | | |  | | | |  |
| Rf& | | | tkfO{sf] ljrf/df Ufe{jtL dlxnfn] s:tf] k\|sf/sf] g'g kof]u ug{‘' k5{< | | ☐v'Nnf jf l9Ss] g'g  ☐jL/] g'g  ☐cfof]l8go'tm g'g  ☐cGo  ☐yfxf ePg | | | | | | !  @  #  $  % | | | |  |
| Rf* | | | ha Ufe{jtL dlxnfn] s'kf]lift x'lG5g, tkfO{sf] ljrf/df s] pgsf] :jf:y hf]lvddf k5{< | | ☐k{5  ☐k{b}g | | | | | | !  ) | | | | olb k{b}g eg] k\|Zg g= r !) ;f]Wg] |
| Rf( | | | olb k5{ eg], :jf:ydf s] s] hf]lvdf b]lVfG5< | | ☐/tmcNktf  ☐Preclampsia (of] cj:yf / o;sf] nI0fsf]af/] a'emfOg] 5_  ☐;qmd0fsf] hf]lvd  ☐d[To'sf] vt/f  ☐cGo | | | | | | !  @  #  $  % | | | | ax' pQ/ |
| Rf!) | | | ha Ufe{jtL dlxnfn] s'kf]lift x'lG5g, tkfO{sf] ljrf/df s] pgsf] lzz'sf] :jf:y hf]lvddf k5{< | | ☐k{5  ☐k{b}g | | | | | | !  ) | | | |  |
| Rf!! | | | tkfO{sf] ljrf/df cfdf s'kf]lift eOg eg] pgsf] gjhft lzz'sf] tf}ndf s:tf] c;/ k5{< | | ☐sd tf}nsf] aRrf -;fgf] aRrf_  ☐d[t aRrf  ☐tf}ndf s'g} c;/ k{b}g  ☐yfxf 5}g | | | | | | !  @  #  $ | | | |  |
| Efu 5 3/fo;L vfB ;'/Iff;DaGwL k\|ZgfjnL | | | | | | | | | | | | | | | |
| 5! | | | ljt]sf] $ xKtfdf s] tkfO{ kl/jf/nfO{ k'Ug] ul/ vfBGg 5}g egL lrlGtt x'g' eof]< | | ☐ lyof]  ☐ lyPg | | | | | | !  ) | | | | olb lyPg eg] k\|Zg g= 5 @ ;f]Wg] |
| 5 !=! | | | ljt]sf] $ xKtfdf o:tf] slt k6s eof]< | | ☐ lj/n} -! b]lv @ k6s_  ☐ slxn]sflx -# b]lv !) k6s_  ☐ ;w}h;f] -!) k6s eGbf al9_ | | | | | | !  @  # | | | |  |
| 5 @ | | | ljt]sf] $ xKtfdf s] tkfO{ jf tkfO{sf] kl/jf/sf] s'g} klg ;b:ox?n] ;fwg ;\|f]tsf] sldn] ubf{ cfkm'nfO{ dgk/]sf] vfg]s'/f vfg gkfpg' ePsf] lyof]< | | ☐ lyof]  ☐ lyPg | | | | | | !  ) | | | | olb lyPg eg] k\|Zg g= 5 # ;f]Wg] |
| 5 @=! | | | ljt]sf] $ xKtfdf o:tf] slt k6s eof]< | | ☐ lj/n} -! b]lv @ k6s_  ☐ slxn]sflx -# b]lv !) k6s_  ☐ ;w}h;f] -!) k6s eGbf al9_ | | | | | | !  @  # | | | |  |
| 5 # | | | jt]sf] $ xKtfdf s] tkfO{ jf tkfO{sf] kl/jf/sf] s'g} klg ;b:ox?n] ;fwg ;\|f]tsf] sldn] ubf{ l;ldt k\|sf/sf vfg]s'/f vfg'k/]sf] lyof]< | | ☐ lyof]  ☐ lyPg | | | | | | !  ) | | | | olb lyPg eg] k\|Zg g= 5 $ ;f]Wg] |
| 5 #=! | | | ljt]sf] $ xKtfdf o:tf] slt k6s eof]< | | ☐ lj/n} -! b]lv @ k6s_  ☐ slxn]sflx -# b]lv !) k6s_  ☐ ;w}h;f] -!) k6s eGbf al9_ | | | | | | !  @  # | | | |  |
| 5 $ | | | ljt]sf] $ xKtfdf s] tkfO{ jf tkfO{sf] kl/jf/sf] s'g} klg ;b:ox?n] ;fwg ;\|f]tsf] sldn] ubf{ cfkm'nfO{ dg gk/]sf] vfg]s'/f vfg'k/]sf] lyof]< | | ☐ lyof]  ☐ lyPg | | | | | | !  ) | | | | olb lyPg eg] k\|Zg g= 5 % ;f]Wg] |
| 5$=! | | | ljt]sf] $ xKtfdf o:tf] slt k6s eof]< | | ☐ lj/n} -! b]lv @ k6s_  ☐ slxn]sflx -# b]lv !) k6s_  ☐ ;w}h;f] -!) k6s eGbf al9_ | | | | | | !  @  # | | | |  |
| 5 % | | | ljt]sf] $ xKtfdf s] tkfO{ jf tkfO{sf] kl/jf/sf] s'g} klg ;b:ox?n] vfgf gePsf] sf/0fn] ubf{ ;fwf/0f cj:yf eGbf sd -vfgfsf] dfqf sd _ vfg'k/]sf] lyof]< | | ☐ lyof]  ☐ lyPg | | | | | | !  ) | | | | olb lyPg eg] k\|Zg g= 5 ^ ;f]Wg] |
| 5 %=! | | | ljt]sf] $ xKtfdf o:tf] slt k6s eof]< | | ☐ lj/n} -! b]lv @ k6s_  ☐ slxn]sflx -# b]lv !) k6s_  ☐ ;w}h;f] -!) k6s eGbf al9_ | | | | | | !  @  # | | | |  |
| 5 ^ | | | ljt]sf] $ xKtfdf s] tkfO{ jf tkfO{sf] kl/jf/sf] s'g} klg ;b:ox?n] ;fljsdf vfg] eGbf sd k6s vfgf vfg'k/]sf] lyof]< | | ☐ lyof]  ☐ lyPg | | | | | | !  ) | | | | olb lyPg eg] k\|Zg g= 5 & ;f]Wg] |
| 5 ^=! | | | ljt]sf] $ xKtfdf o:tf] slt k6s eof]< | | ☐ lj/n} -! b]lv @ k6s_  ☐ slxn]sflx -# b]lv !) k6s_  ☐ ;w}h;f] -!) k6s eGbf al9_ | | | | | | !  @  # | | | |  |
| 5 & | | | ljt]sf] $ xKtfdf s] tkfO{x?n] ;fwg ;\|f]tsf] sldn] ubf{ vfg]s'/f ;lsP/ klg vfgf lsGg g;Sg'ePsf]lyof]< | | ☐ lyof]  ☐ lyPg | | | | | | !  ) | | | | olb lyPg eg] k\|Zg g= 5 * ;f]Wg] |
| 5 &=! | | | ljt]sf] $ xKtfdf o:tf] slt k6s eof]< | | ☐ lj/n} -! b]lv @ k6s_  ☐ slxn]sflx -# b]lv !) k6s_  ☐ ;w}h;f] -!) k6s eGbf al9_ | | | | | | !  @  # | | | |  |
| 5 * | | | ljt]sf] $ xKtfdf s] tkfO{ jf tkfO{sf] kl/jf/sf] s'g} klg ;b:ox?n] kof{Kt vfg]s'/f gePsf] sf/0fn] ubf{ slxNo} /ftL ef]s} ;'Tg'k/]sf] lyof]< | | ☐ lyof]  ☐ lyPg | | | | | | !  ) | | | | olb lyPg eg] k\|Zg g= 5 ( ;f]Wg] |
| 5*=! | | | ljt]sf] $ xKtfdf o:tf] slt k6s eof]< | | ☐ lj/n} -! b]lv @ k6s_  ☐ slxn]sflx -# b]lv !) k6s_  ☐ ;w}h;f] -!) k6s eGbf al9_ | | | | | | !  @  # | | | |  |
| 5 ( | | | ljt]sf] $ xKtfdf s] tkfO{ jf tkfO{sf] kl/jf/sf] s'g} klg ;b:ox?n] ;fwg ;\|f]tsf] sldn] ubf{ slxNo} lbg / /fte/ ef]s} gvfOsg a:g'k/]sf] lyof]< | | ☐ lyof]  ☐ lyPg | | | | | | !  ) | | | | olb lyPg eg] k\|Zg g= h !;f]Wg] |
| 5 (=! | | | ljt]sf] $ xKtfdf o:tf] slt k6s eof]< | | ☐ lj/n} -! b]lv @ k6s_  ☐ slxn]sflx -# b]lv !) k6s_  ☐ ;w}h;f] -!) k6s eGbf al9_ | | | | | | !  @  # | | | |  |
| Efu h dlxnf ;zlSts/0f ;DaGwL hfgsf/L | | | | | | | | | | | | | | | |
| Hf! | | | s] tkfO{ s'g} ;d'xdf ;+nUg x'g' x'G5<  cfdf ;d"x  ;xsf/L÷jrt ;d"x  dlxnf ;d"x | | ☐ 5 ☐ 5}g  ☐ 5 ☐ 5}g  ☐ 5 ☐ 5}g  ☐ cGo -pNn]v ug]{_================= | | | | | | 5 Ö!  5}gÖ) | | | |  |
| Hf@ | | | s] of] 3/ jf s'g} csf{] 3/, tkfO{sf] gfddf Psn jf ;+o"Qm?kdf 5< | | ☐ cfkm" PSn}sf]  ☐ ;+o"Qm  ☐ b'a} Psn / ;+o"Qm?kdf  ☐ cfˆgf] gfddf gePsf] | | | | | | !  @  #  $ | | | |  |
| Hf# | | | s] s'g} hUuf, tkfO{sf] gfddf Psn jf ;+o"Qm?kdf 5< | | ☐ cfkm" PSn}sf]  ☐ ;+o"Qm  ☐ b'a} Psn / ;+o"Qm?kdf  ☐ cfˆgf] gfddf gePsf | | | | | | !  @  #  $ | | | |  |
| Hf$ | | | tkfO{sf] :jf:Yo :ofxf/ ;DaGwL lg0f{o s;n] ug'{ x'G5< | | ☐ cfkm" PSn}sf]  ☐ ;+o"Qm  ☐ b'a} Psn / ;+o"Qm?kdf  ☐ cfˆgf] gfddf gePsf | | | | | | !  @  #  $ | | | |  |
| Hf% | | | tkfO{sf] kl/jf/sf] k\|d'v 3/fo;L lsgd]n ;DaGwL lg0f{o s;n]ug'{ x'G5< | | ☐ cfkm" PSn}sf]  ☐ ;+o"Qm  ☐ b'a} Psn / ;+o"Qm?kdf  ☐ cfˆgf] gfddf gePsf | | | | | | !  @  #  $ | | | |  |
| Hf^ | | | tkfO{sf] kl/jf/ jf gft]bf/;uFsf] e]6sf] lg0f{o s;n] ug'{ x'G5< | | ☐ cfkm" PSn}sf]  ☐ ;+o"Qm  ☐ b'a} Psn / ;+o"Qm?kdf  ☐ cfˆgf] gfddf gePsf | | | | | | !  @  #  $ | | | |  |
